# Palatal Congenital Melanocytic Nevus With Unusual Clinical Feature: A Rare Case Report With Literature Review

**DOI:** 10.1002/ccr3.73101

**Published:** 2026-07-06

**Authors:** Saede Atarbashi‐Moghadam, Ali Lotfi, Mohammadreza Kashefi Baher

**Affiliations:** ^1^ Department of Oral and Maxillofacial Pathology, School of Dentistry Shahid Beheshti University of Medical Sciences Tehran Iran; ^2^ Health Research Center Chamran Hospital Tehran Iran

**Keywords:** biopsy, congenital, melanocytes, palate, pigmented nevus

## Abstract

Congenital melanocytic nevi are uncommon benign lesions that primarily involve the skin and are exceedingly rare within the oral cavity, where their atypical presentation may pose a diagnostic challenge due to clinical overlap with other pigmented lesions. We report a case of an intramucosal palatal congenital melanocytic nevus (CMN) in a 34‐year‐old woman who presented with a painless nodular lesion arising from a lifelong pigmented area of the palate. Histopathological examination demonstrated a benign proliferation of nevus cells with theques formation. The deepest nevus cells showed proximity to the adjacent minor salivary gland lobules. The lesion was managed by complete surgical excision, with uneventful healing at 6‐month follow‐up. A comprehensive review of the literature identified only 11 reported cases of intraoral CMNs, confirming the palate as the most frequently involved site and a marked female predominance. This case highlights the critical role of correlating clinical history with histopathological findings to achieve an accurate diagnosis.

## Key Clinical Message

Congenital melanocytic nevi are uncommon, and their intraoral presentation is exceptionally rare, with only 12 cases, including the present case, reported in the English‐language literature. Accurate diagnosis requires a comprehensive, integrated evaluation of the patient's history, clinical features, and histopathologic findings, with biopsy being essential to definitively exclude melanoma.

## 1 Introduction

Melanocytic nevi are benign proliferations of cells derived from melanocytic cells of neural crest origin with unknown etiology that may be either acquired or congenital [1, 2]. These lesions predominantly affect cutaneous regions and are rarely encountered in the oral mucosa, with an estimated annual incidence of approximately 4.35 cases per 10 million individuals and a prevalence of up to 0.5% in the oral cavity of the general population [1, 2, 3].

Congenital melanocytic nevus (CMN) is a less common subtype of melanocytic nevi that presents at birth or shortly thereafter, arising from the accumulation of melanocytes in ectopic locations [4, 5]. These lesions can affect up to 2% of newborns [6]. Clinically, congenital nevi are usually solitary lesions [7] that may present with a papular, pebbly, verrucous, or hypopigmented appearance [8]. Although CMNs usually present on the trunk, their manifestation in the oral cavity is exceptionally uncommon [9].

Previously reported cases of oral CMNs demonstrate a marked female predominance, with a female‐to‐male ratio of 3:1 (Table S1). Although the age at referral varies among patients, the congenital nature of these lesions implies a relatively consistent age at initial presentation [2]. Moreover, the limited epidemiological, clinical, and histopathological data on oral CMNs add to their diagnostic complexity.

This case report documents an intramucosal CMN of the palate, underscoring its uncommon clinical presentation, demographic context, and characteristic histopathology. It also incorporates a comprehensive review of the oral CMNs literature to broaden the currently limited understanding of this rare entity.

## 2 Case History/Examination

The patient was a 34‐year‐old woman referred to a private pathology center (Tehran, Iran) in June 2025 for evaluation of a painless mass on the posterior right palate. The patient reported a flat, uniformly brown palatal pigmentation present since birth, noting that it was initially smaller, approximately 0.5 cm in diameter, according to her recollection. Following a palatal burn sustained after ingesting a hot liquid 6 months earlier, the patient noticed enlargement of the lesion during self‐examination and subsequently sought medical evaluation.

**FIGURE 1:**
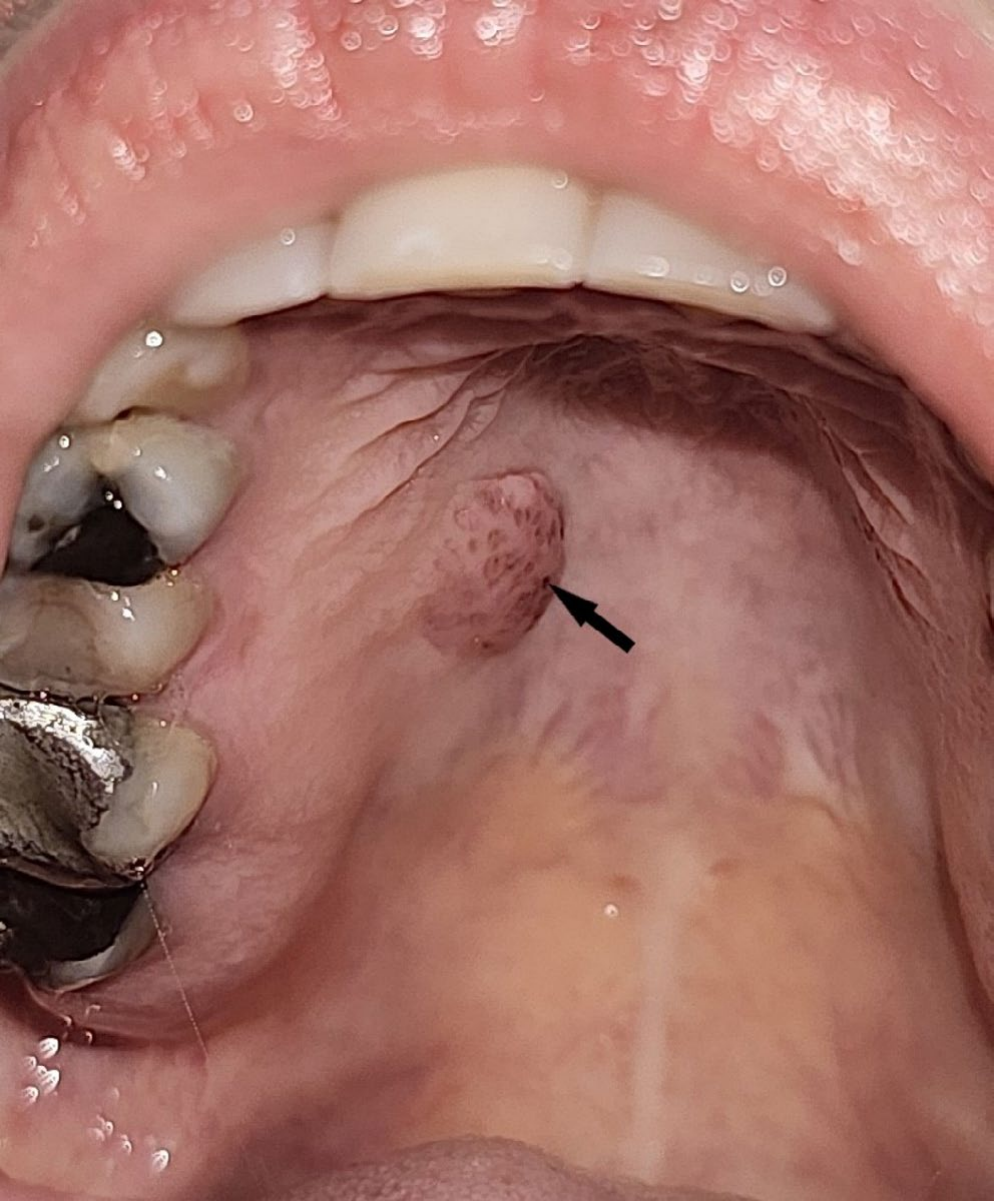
Clinical photograph showing an exophytic, sessile nodular mass with normal mucosal coloration and scattered brown spots (black arrow), resulting in a speckled appearance.

Intraoral examination revealed an exophytic sessile nodular mass with a soft consistency, measuring 1.5 cm in its greatest diameter. The lesion was covered by normal mucosa with an irregular surface and scattered brown spots, giving it a speckled appearance (Figure 1). All other intraoral sites appeared unremarkable. Extraoral examinations revealed no abnormalities, and laboratory findings were within normal limits. The orthopantomogram revealed no abnormalities, with no evidence of any intraosseous lesions.

**FIGURE 2:**
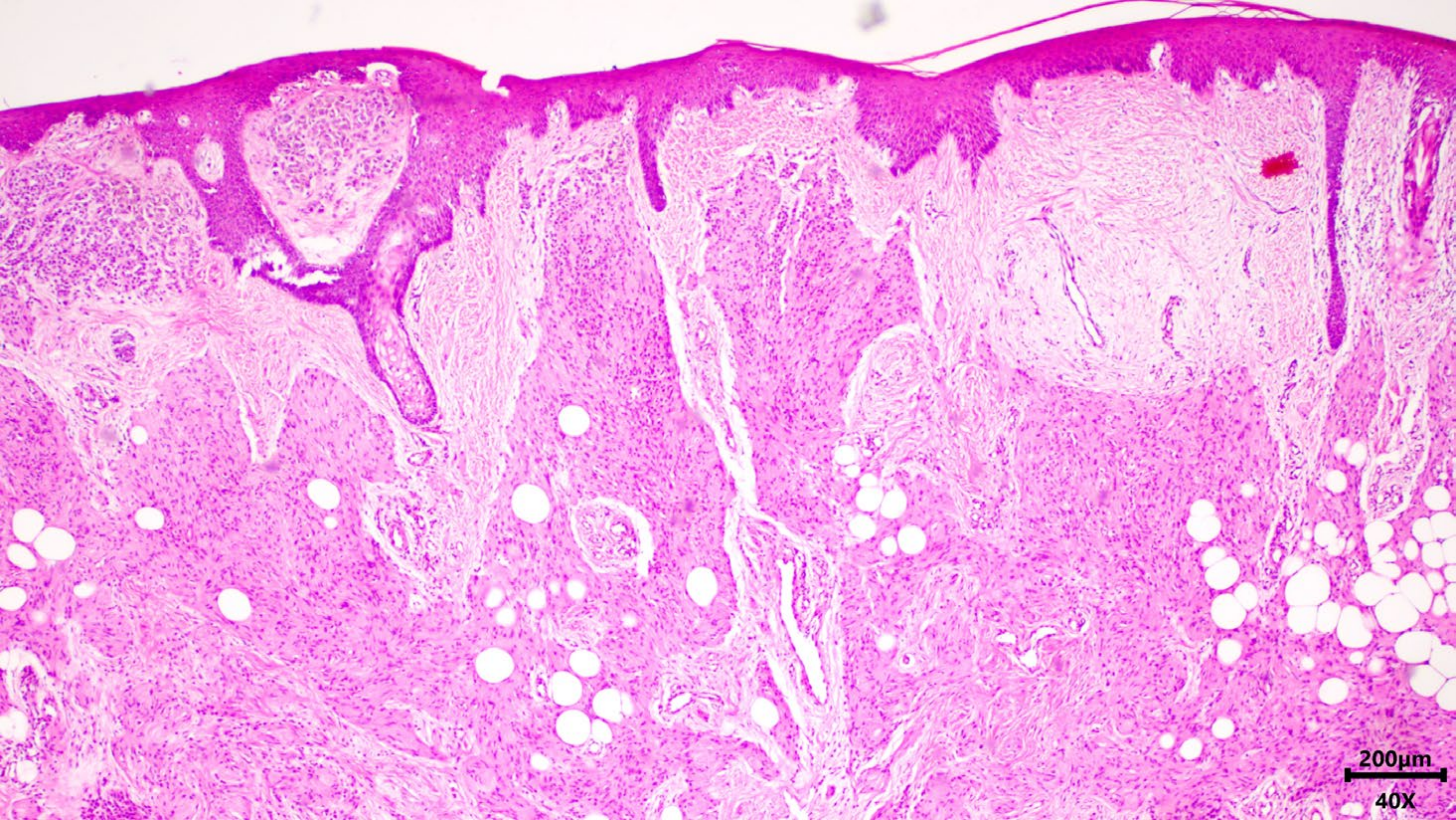
Histopathologic section showing nevus cell proliferation in the underlying connective tissue. Superficial nevus cells appear ovoid, whereas deeper cells exhibit spindle morphology. The lesion is covered by keratinized stratified squamous epithelium (H&E, ×40).

**FIGURE 3:**
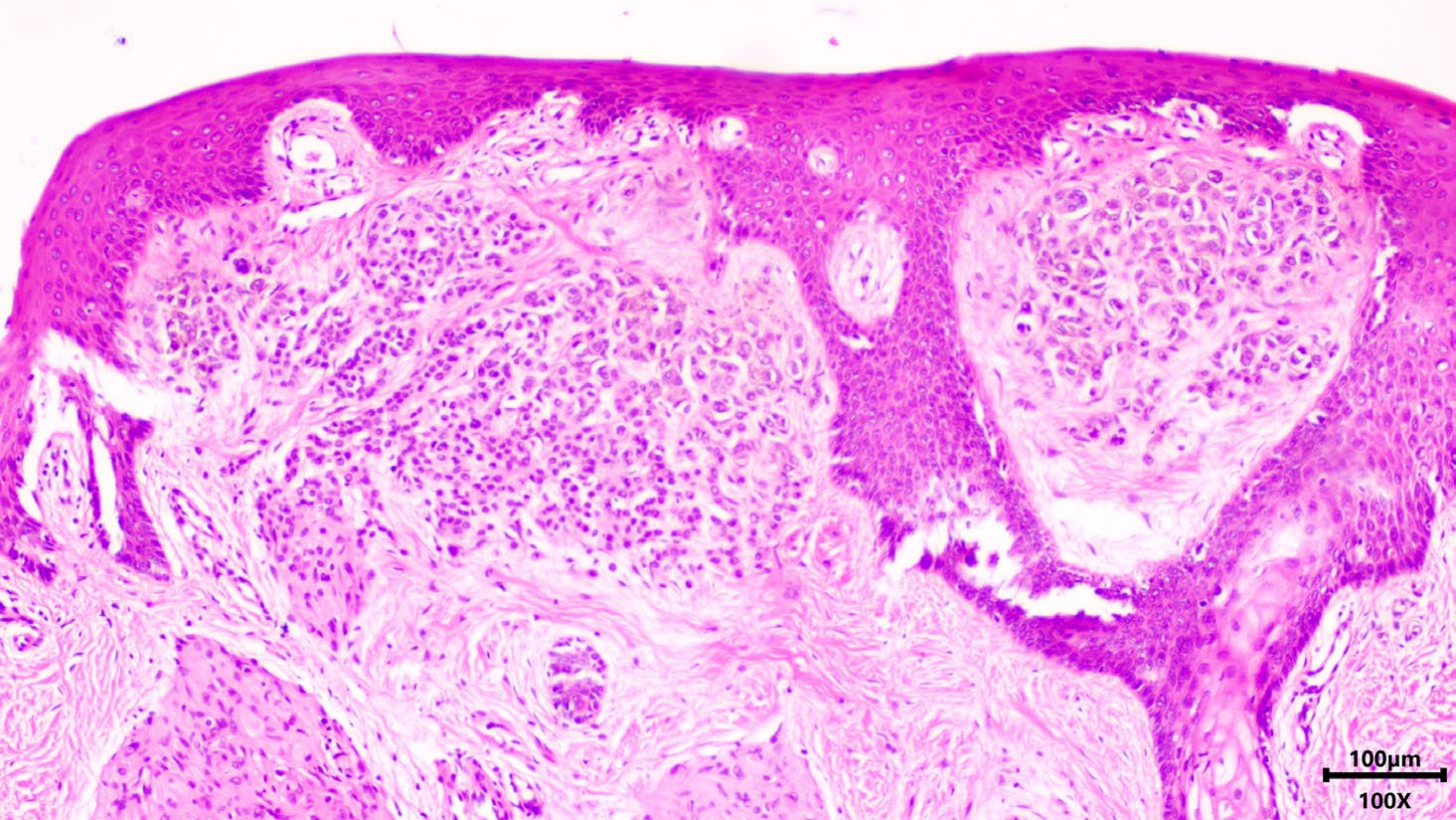
Histopathologic section illustrating superficial plump ovoid cells with theque formation. No melanin pigmentation is observed within the basal cell layer (H&E, ×100).

**FIGURE 4:**
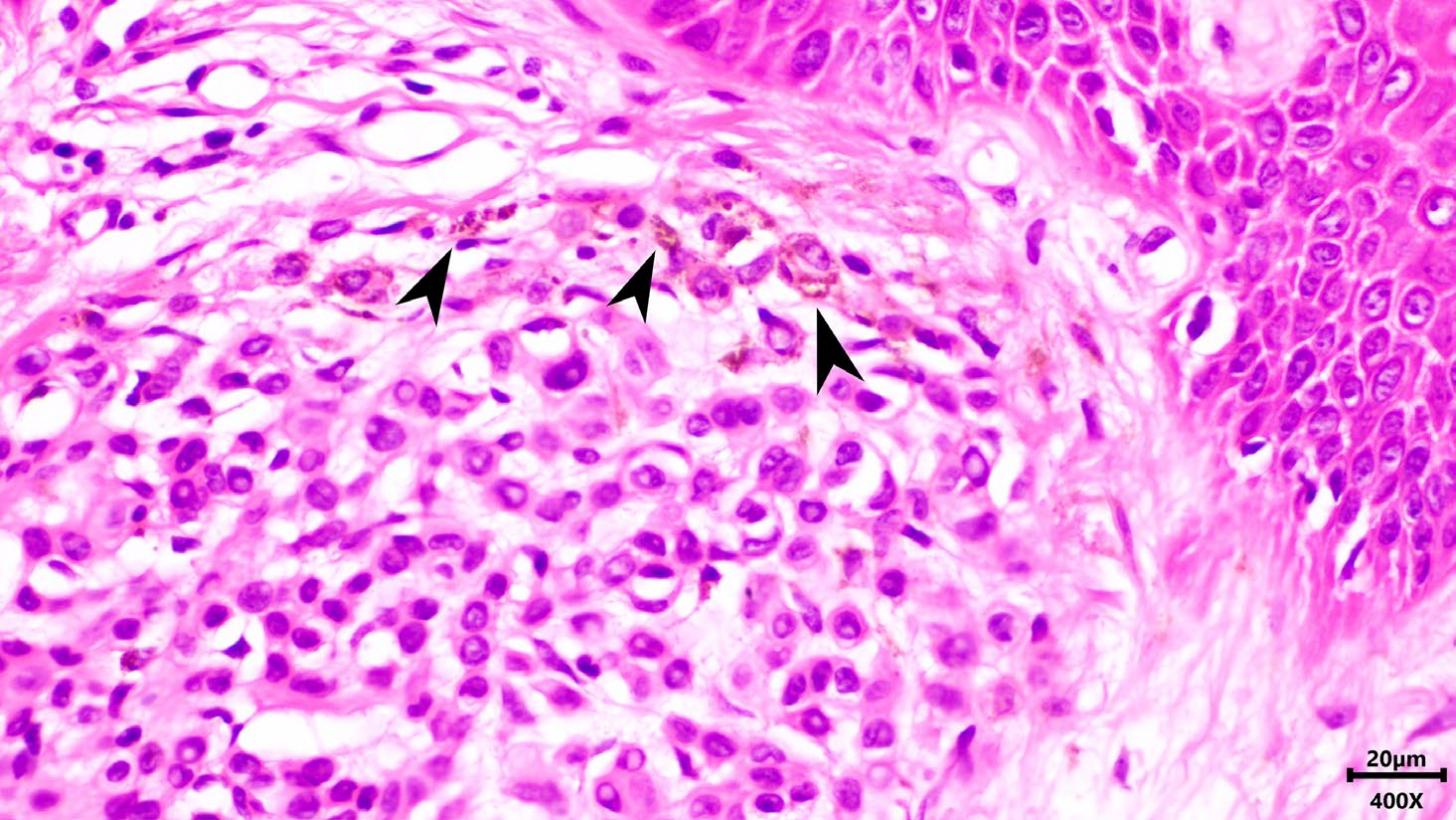
Histopathologic section demonstrating ovoid to epithelioid cells with scattered melanin pigmentation in a patchy distribution (black arrowheads) (H&E, ×400).

To obtain a definitive diagnosis, an excisional biopsy was performed under local anesthesia, with a provisional diagnosis of oral CMN, given the reported lifelong duration of the pigmentation. The specimen showed a solid, white, and homogenous cut surface with scattered brown spots, which was processed for Hematoxylin and Eosin (H&E) histopathologic study. Microscopic examination revealed a benign, unencapsulated proliferation of ovoid to epithelioid cells with abundant cytoplasm (nevus cells) with theques formation (nests of nevus cells). No melanin was observed in the basal layer of the overlying epithelium. However, limited, sparse melanin deposition was identified in the superficial cells of the lamina propria, presenting in a focal and patchy pattern. No evidence of inflammation or surface epithelial ulceration was observed. The deepest nevus cells appeared elongated and spindle‐shaped, lacked pigmentation, and showed proximity to the adjacent minor salivary gland lobules. The overlying epithelium showed an irregular surface (Figures 2, 3, 4).

**FIGURE 5:**
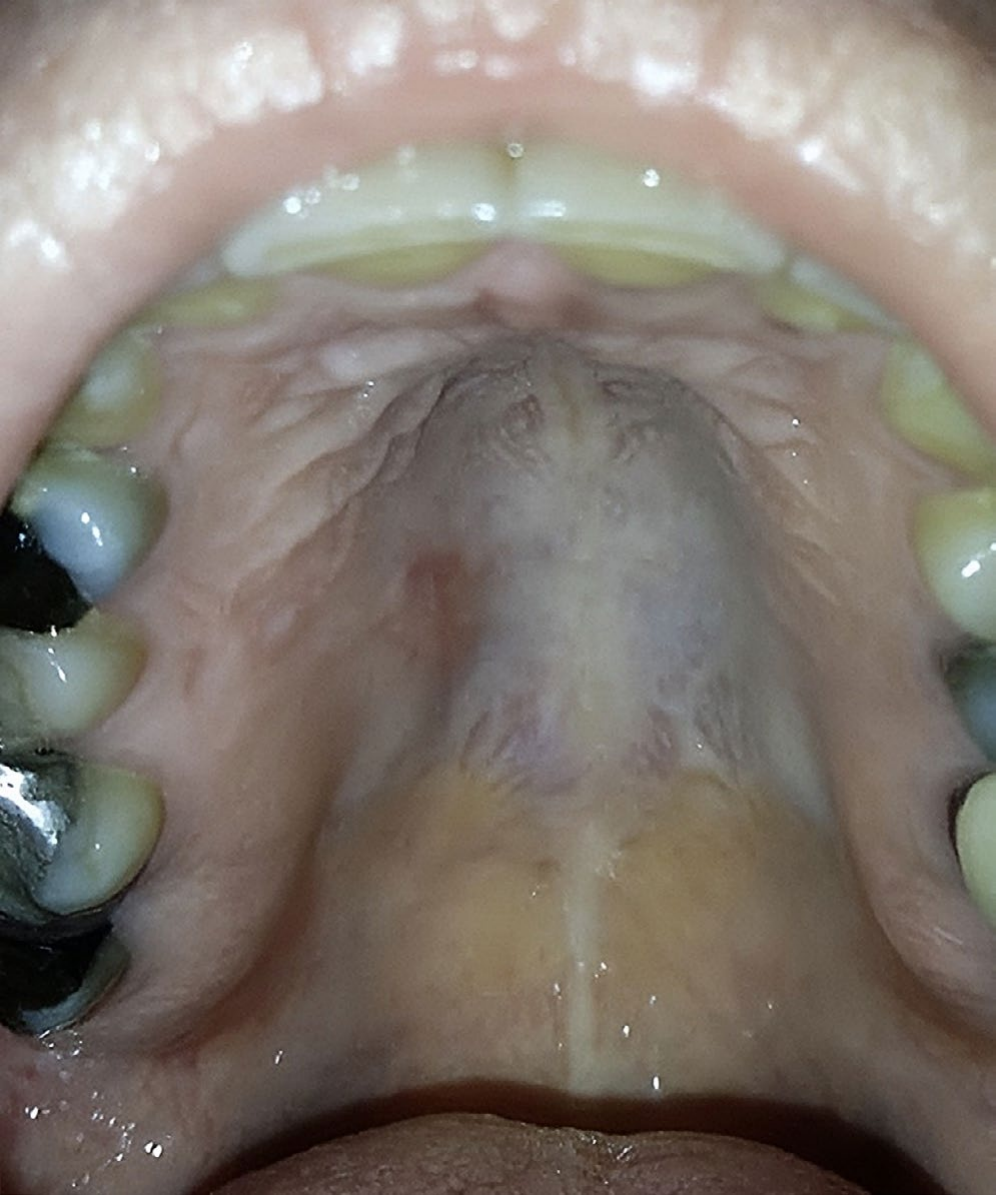
Uneventful healing of the surgical site with satisfactory outcomes at six‐month follow‐up.

The histopathological features, along with the patient's past medical history and clinical findings, were consistent with the diagnosis of oral CMN (intramucosal type). The patient remains under regular annual follow‐up, and at 6 months, the surgical site demonstrated uneventful healing with satisfactory outcomes (Figure 5).

## 3 Differential Diagnosis, Investigations, and Treatment

Possible differential diagnoses for the present case, given the presence of scattered brown pigmentation within the lesion, included oral melanocytic nevi. However, the lifelong persistence of the pigmentation supports a diagnosis of CMN. Additionally, given the palatal location and the peripheral nature of the lesion, supported by the absence of radiographic changes on orthopantomogram, other differential diagnoses included salivary gland tumors and benign soft tissue neoplasms. Malignant melanoma was excluded based on the lesion's well‐circumscribed margins and its lifelong presence.

Investigations included a comprehensive medical history, thorough intra‐ and extraoral clinical examinations, and relevant paraclinical assessments.

## 4 Outcome and Follow‐Up

Oral CMNs represent an exceptionally rare entity, with a challenging diagnosis due to their extremely low incidence, broad clinical heterogeneity, and significant overlap with other intraoral lesions. The present case of an intramucosal palatal CMN in an adult female highlights the critical role of integrating a lifelong clinical history with careful histopathological evaluation to establish an accurate diagnosis. Furthermore, since these lesions cannot be reliably distinguished from their differential diagnoses on clinical grounds alone, biopsy remains essential. Moreover, any change in lesion characteristics should prompt a timely histopathological assessment, complemented by annual clinical examination. Complete surgical excision, serving as both the definitive diagnostic and therapeutic approach, resulted in uneventful healing at six‐month follow‐up, with no evidence of recurrence.

## 5 Discussion

### 5.1 Case Context and Review of the Literature

This case illustrates a rare palatal CMN in a 34‐year‐old female, presenting with atypical clinical features that highlight the need for careful evaluation and exclusion of differential diagnoses. Given the rarity of this lesion as an intraoral mass, available demographic, clinical, and histopathological data remain limited. To bridge this gap, a comprehensive literature search was undertaken across PubMed, Scopus, and Web of Science, employing the query (Oral OR Mouth) AND (Congenital) AND (Nevus OR Nevi), which identified 11 well‐documented English‐language cases of oral CMN. Table S1 summarizes the demographic, clinical, and histopathological characteristics of reported intraoral CMNs, as well as their management and follow‐up intervals.

### 5.2 Demographic Characteristics

Regarding the demographic profile, the present case is consistent with the female predominance identified in the literature review but differs in demonstrating a higher age at presentation compared with the calculated mean of 21.3 years. It should be acknowledged that the congenital origin of the lesion implies its presence prior to referral, while the timing of referral itself may vary depending on patient‐specific factors. In this case, referral was prompted by the patient's self‐examination after experiencing prior burning in the same area, which led to care‐seeking for the painless palatal mass.

### 5.3 Anatomical Site

Palatal involvement in the present case aligns with findings from the literature review, which identifies the palate as the most common site of intraoral CMNs, followed by the labial mucosa, upper lip, gingiva, and buccal mucosa. While most reported lesions are solitary, rare instances of multifocal intraoral involvement have been documented [2, 9]. The delayed detection of palatal CMN observed in this study likely reflects the inherently limited visual accessibility of this anatomic region. However, a palatal CMN case has been reported in which the patient's chief complaint was a bothersome sensation due to progressive surface roughness, in the absence of pain or dysphonia [8].

### 5.4 Lesion Size

The 1.5‐cm lesion observed in the present case, as well as the dimensions reported for other oral CMNs in the literature (mean 1.86 ± 1.34 cm; range 0.7–5.0 cm), exceed the established 0.6‐cm cutoff—below which lesions are typically classified as acquired nevi and above which they are considered congenital—thereby supporting the congenital nature of the lesion [6]. In addition, unlike acquired nevi, which may undergo regression, congenital nevi typically persist over time [10]. This is consistent with the lifelong persistence of the lesion observed in the present case.

### 5.5 Hypopigmented Appearance

Although oral CMNs have been reported to exhibit a broad spectrum of colors (Table S1), the present case demonstrated an unusual clinical presentation characterized by normal mucosal coloration interspersed with scattered brown spots, resulting in a speckled appearance (Figure 1). The authors propose that this pattern may be described as a hypopigmented variant. This interpretation is supported by three key features of the present case. First, according to the patient's history, the brown palatal pigmentation was initially uniform in color; however, by the time of referral, only scattered brown spots remained, suggesting a reduction in lesion pigmentation. Second, the observed white cut surface further corroborates this clinical presentation. Third, the reduced melanin content exhibited regional variation in density and a patchy distribution pattern, confined exclusively to the epithelioid and ovoid cells within the lamina propria, corresponding to the clinical appearance. Similarly, hypopigmented or amelanotic papules have been reported as acquired oral melanocytic nevi, complicating clinical recognition and highlighting the importance of histopathological examination for definitive diagnosis [11, 12]. Based on current evidence, this appears to be the first reported case of an oral CMN presenting with a hypopigmented appearance. This finding may be further explained by the evolutionary stages of intraoral nevi (junctional, compound, and intramucosal), whether congenital or acquired, whereby progressive extension into the connective tissue results in reduced visibility of melanin, leading to a hypopigmented presentation [2, 10].

### 5.6 Thermal Injury and Potential Mechanisms

Trauma to the oral soft tissues is well established as a cause of reactive hyperplasia and mucosal overgrowth. Consistent with the present case, thermal injuries most commonly arise from accidental ingestion of hot foods or beverages and typically involve the palate [13]. However, histopathological examination revealed no evidence of inflammation, and the epithelium remained intact without ulceration, making a reactive process (trauma‐induced proliferation) an unlikely explanation. In this context, the reported palatal burn may represent a coincidental finding that merely increased the patient's awareness, ultimately leading to detection of the lesion. To the best of the authors' knowledge, no published studies support an association between thermal injury and nevus evolution. Nevertheless, this case may prompt further investigation into this potential relationship, with particular consideration of mechanisms such as reactive hyperplasia, vascular alterations, or edema.

### 5.7 Extraoral Involvement

Although no extraoral involvement was observed in the present case, such manifestations have been reported in various anatomical regions, such as the trunk, palms, soles, lower back/lumbar region [7], inner thigh [8], chin, lower costal margin [9], nasal sill, oral commissure, philtral columns [9], eyes, and the scapular/infrascapular area [14].

### 5.8 Differential Diagnosis

A broad spectrum of differential diagnoses must be considered, including various pigmented oral lesions, exogenous pigmentation (amalgam tattoo), benign and malignant melanocytic neoplasms such as acquired melanocytic nevus, neuroectodermal tumors, resolving submucosal hematoma, melanotic macule (focal benign melanosis), physiological pigmentation, smoker's melanosis, malignant melanoma, and melanoacanthoma [1, 6, 8, 15]. Furthermore, the specific clinical characteristics observed in the current study extend this list to include salivary gland tumors and benign soft‐tissue tumors. It is worth mentioning that ill‐defined borders have also been reported in a large oral CMN, which further complicates its diagnosis and differentiation from malignant melanoma [2].

### 5.9 Histopathological Features and Diagnostic Approach

The histopathological characteristics of oral and cutaneous melanocytic nevi may exhibit considerable overlap [1, 6]. CMN typically shows a diffuse band‐like or sheet‐like infiltrate of nevus cells streaming through collagen bundles, blood vessels, nerves, or salivary ducts, demonstrating a deeper extension than the classic nest‐like architecture characteristic of acquired nevi [2, 6, 8]. This is consistent with the present study, which demonstrated proximity to the adjacent minor salivary gland lobules. Additionally, Torres et al. [9] reported a progressive reduction in both cytoplasmic volume and pigment content of lesional cells as the lesion extended from the lamina propria into the submucosa, with minimal melanin deposition observed in the basal layer. Similarly, the deepest nevus cells were devoid of pigmentation in the present case.

Overall, the distinction of a congenital, rather than an acquired, nevus in the present case is based on the integrated assessment of lifelong persistence, larger lesion size, and characteristic histopathological features. These criteria are consistent with those proposed by Marangon Júnior et al. [2]. However, lacking information about the presence of the lesion at birth does not exclude a congenital nature, as the detection of intraoral masses in newborns remains a central diagnostic challenge.

It is worth noting that a diagnostic biopsy is generally warranted for any unexplained intraoral pigmented lesion to exclude melanoma [1, 6, 8]. However, Bracamonte et al. [15] stated that oral melanocytic nevi do not require treatment when the diagnosis is clinically reliable and other concerning etiologies have been excluded. Moreover, Gilbert et al. [8] reported that small CMNs can be managed conservatively with clinical follow‐up. Given the more aggressive course of oral malignant melanoma compared with its cutaneous counterpart, any alteration within an intraoral CMN should be regarded as suspicious and prompt a timely biopsy rather than prolonged observation [9].

Various immunohistochemical (IHC) markers, including MART‐1, HMB‐45, Ki‐67, FASN, S‐100, bcl‐2, and p16, have been employed in the characterization of reported oral CMNs [2, 4, 8]. However, the characteristic histopathological features observed in this case render additional IHC evaluation unnecessary.

### 5.10 Management

Complete surgical excision, as performed in the present case, is the preferred treatment, as it minimizes morbidity and allows for thorough histopathological evaluation [1, 2, 8]. However, treatment decisions should be individualized, considering lesion size, anatomic constraints, and patient‐specific factors such as comorbidities and ability to adhere to follow‐up [8]. Management strategies for documented oral CMNs range from observation without intervention [7, 14, 15] to incisional [2, 8, 9] and excisional biopsy [4, 5, 6, 8, 16, 17]. Moreover, involvement of the upper lip warrants particular caution because of its aesthetic significance and may require combined local flaps with a modified triangular incision in a plastic surgery setting [5, 17]. Keloid formation in this region may also necessitate adjunctive cryosurgery [5].

### 5.11 Follow‐Up and Long‐Term Risks

The mean follow‐up duration among oral CMNs with sufficient available data was 2.72 ± 4.17 years (range: 4 months to 11 years). The six‐month follow‐up period represents the main limitation of the current study; however, the patient will be monitored on a regular annual schedule. Given the lesion's low but existing potential for recurrence or malignant transformation, and notably, one reported case recurred after 3 years [4], the authors recommend long‐term surveillance. Overall, the risk of malignant transformation in the oral melanocytic nevi is considered negligible [1, 3], whereas cutaneous CMNs carry an elevated risk of melanoma development, with this risk appearing to correlate with lesion size [2, 4]. In addition, a more focused call is recommended for standardized reporting of oral CMNs by dentists and oral pathologists as first‐line identifiers, in order to advance understanding of clinicopathological behavior and inform evidence‐based diagnostic and management strategies for this rare entity.

## Author Contributions

**Saede Atarbashi‐Moghadam:** conceptualization, investigation, supervision, writing – review and editing. **Ali Lotfi:** investigation, methodology, validation, visualization. **Mohammadreza Kashefi Baher:** conceptualization, data curation, formal analysis, investigation, methodology, supervision, visualization, writing – original draft, writing – review and editing.

## Funding

The authors have nothing to report.

## Ethics Statement

The authors have nothing to report.

## Consent

Written informed consent for the publication of this case, including all clinical details and any accompanying images, was obtained from the patient.

## Conflicts of Interest

The authors declare no conflicts of interest.

## Supporting information


**Table S1:** Demographic, clinical, histopathologic, and management characteristics of documented oral CMNs.

## Data Availability

The dataset analyzed during the current study is available from the corresponding author upon reasonable request.
