# Supplementary material for: Palatal Congenital Melanocytic Nevus With Unusual Clinical Feature: A Rare Case Report With Literature Review
Source: Clin Case Rep. 2026 Jul 6;14(7):e73101. doi: 10.1002/ccr3.73101 (PMC13336060; doi:10.1002/ccr3.73101)
Supplement: Supplementary file 1 — Table S1: Demographic, clinical, histopathologic, and management characteristics of documented oral CMNs. [file CCR3-14-e73101-s001.docx]

| **Supplementary Table 1. Demographic, clinical, histopathologic, and management characteristics of documented oral CMNs** | | | | | | | | | | |
| --- | --- | --- | --- | --- | --- | --- | --- | --- | --- | --- |
| **Author/Year** | **Age /Sex** | **Location** | **Color** | **Size (CM)** | **Extraoral Involvement** | **Differential Diagnosis** | **Histopathologic Findings** | **IHC markers** | **Management** | **Follow-up** |
| **Takeda et al./**  **1988 (16)** | 7/F | Maxillary labial mucosa | Brownish black | 0.7 | None | NA | Intramucosal nests of ovoid-shaped nevus cells, extending from the lamina propria to deep submucosal connective tissue.  Scattered multinucleated giant cells. | None | Excisional biopsy | 3 Years |
| **Allen et al./**  **1995 (6)** | 3/F | Mandibular lingual gingiva | Dark brown | 1.5 | None | Melanocytic nevus, melanotic neuroectodermal tumor of infancy, exogenous pigmentation, resolving submucosal hematoma, melanoma | Intramucosal sheets of melanocytes extend to the deep margin of the sample.  Melanin deposits. | None | Excisional biopsy | 6 Months |
| **Rose et al./**  **2003 (7)** | 19/F | Buccal mucosa | NA | NA | Trunk, face, palms, soles, lower back | NA | None | None | None | NA |
| **Gilbert et al./**  **2011 (8)** | 19/F | Hard palate | Erythematous with scattered light brown pigmentation | 1.2 | History of excised inner thigh CMN | Benign and malignant melanocytic neoplasms | Intramucosal sheets of mature nevus cells extend deep in the lamina propria and around salivary gland ducts and nerves.  Melanin deposits.  Scattered multinucleated giant cells; papillomatous oral mucosa epithelium. | Melan A: +  HMB-45: +  Ki-67: + | Incisional biopsy followed by excisional biopsy | NA |
| **Marangon Júnior et al./**  **2015 (2)** | 16/F | Buccal mucosa,  alveolar mucosa, retromolar triangle | Normal mucosal coloration to scattered brown-to-black pigmentations | 5 | None | Malignant melanoma | Diffuse intramucosal infiltrate of small monomorphous melanocytes, arranged in a band-like pattern.  Splaying of melanocytes between collagen fibers.  No melanin in basal cells or melanocytic hyperplasia. | HMB-45: -  FASN: +  S-100: +  bcl-2: +  Ki-67: + | Incisional biopsy | 11 Years |
| **Salcines et al./**  **2017 (4)** | 9/F | Hard and soft palate | Normal mucosal coloration | 2 | None | NA | Diffuse infiltrate of small epithelioid melanocytes in the lamina propria with involvement of the vessel walls.  Melanocytes are arranged in clusters, theques or bands, streaming through collagen bundles with occasional melanin deposition.  The overlying mucosa exhibited slight papillomatosis with junctional activity. | MART-1: +  p16: +  HMB-45: +  MIB-1: + | The initial excisional biopsy resulted in recurrence after 3 years; the second excisional biopsy was successful | 1 Year |
| **Torres et al./**  **2017 (9)** | 19/M | Mandibular buccal gingiva,  buccal vestibule,  labial mucosa | Normal mucosal coloration to pale white and light brown. | NA | History of excised congenital nevus on the chin, which extended intraorally to the tongue, birth mark on the lower costal margin | NA | Diffuse infiltrate of melanocytes between collagen bundles, containing varying quantities of melanin pigment.  Numerous nests or theques (small, round aggregates) of pigmented epithelioid cells with vesicular nuclei, abundant eosinophilic, glassy cytoplasm, and intracellular melanin were present in the lamina propria.  Perivascular distribution of melanocytes. | None | Incisional biopsy | NA |
| **Meng et al./**  **2018 (17)** | 22/F | Upper lip | Brown | 2 | Nasal sills, commissure, philtral columns | NA | None | None | Excisional biopsy (with two previous partial excisions) | 4 Months |
| **Hellmeister et al./**  **2022 (5)** | 32/F | Upper lip | Dark brown | 1 | None | Malignant melanoma | NA | None | Excisional biopsy | NA |
| **Bracamonte et al./**  **2023 (15)** | 44/M | Tongue | Dark brown | NA | None | Physiological pigmentation, smoker’s melanosis, amalgam tattoo, malignant melanoma | None | None | None | NA |
| **Pillai et al./**  **2024 (14)** | 32/M | Hard palate | Blue | NA | Face, sclera, back, periorbital and malar regions, cheeks, temples, forehead, lumbar and infrascapular areas, scapular region | NA | None | None | None | NA |
| **Present case** | 34/F | Hard palate | Normal mucosal coloration with scattered brown spots (speckled) | 1.5 | None | Salivary gland tumors, benign soft tissue tumors | Unencapsulated proliferation of ovoid to epithelioid cells with abundant cytoplasm (nevus cells) with theques formation and frequent melanin pigmentation. The deepest nevus cells appeared elongated and spindle-shaped, devoid of pigmentation. | None | Excisional biopsy | 6 Months |
| Abbreviations: M (Male) / F (Female) / CM (centimeter) / IHC (Immunohistochemical) / CMN (Congenital Melanocytic Nevus) / FASN (Fatty Acid Synthase) / bcl-2 (B-cell Lymphoma 2) / HMB-45 (Human Melanoma Black-45) / MART-1 (Melanoma Antigen Recognized by T-cells 1)  NA = not available from the original publication because the related data were not reported by authors.  None = explicitly stated as absent or not performed.  Note that the size of the lesion has been reported according to its greatest dimension. | | | | | | | | | | |

**References**

2. Marangon Júnior H, Souza PEA, Soares RV, de Andrade BAB, de Almeida OP, Horta MCR. Oral congenital melanocytic nevus: a rare case report and review of the literature. Head and neck pathology. 2015;9(4):481-7.

4. Salcines A, Woo S, Noonan V, Mansfield M, Li C. Rare Intraoral Congenital Melanotic Nevus-A Case Report. Oral surgery, oral medicine, oral pathology and oral radiology. 2017;124(3):e220-e1.

5. Hellmeister L, Agatti L, Prata D, Gimenez R, Toth T, Montalli V, Moraes P. Congenital Labial Nevus Treated With Plastic Surgery and Cryosurgery. Oral Surgery, Oral Medicine, Oral Pathology and Oral Radiology. 2022;134(3):e136.

6. Allen CM, Pellegrini A. Probable congenital melanocytic nevus of the oral mucosa: case report. Pediatric dermatology. 1995;12(2):145-8.

7. Rose C, Kaddu S, El-Sherif TF, Kerl H. A distinctive type of widespread congenital melanocytic nevus with large nodules. Journal of the American Academy of Dermatology. 2003;49(4):732-5.

8. Gilbert ML, Hanna W, Ghazarian D, Dover D, Klieb HB. Congenital melanocytic nevus of the oral mucosa: report of a rare pigmented lesion and review of the literature. Clinics and practice. 2011;1(1):e17.

9. Torres KG, Carle L, Royer M. Nevus Spilus (Speckled Lentiginous Nevus) in the Oral Cavity: Report of a Case and Review of the Literature. Am J Dermatopathol. 2017;39(1):e8-e12.

14. Pillai NS, Buccha Y, Nair RS, Kothari R, Malik N. Bilateral Naevus of Ito and Ota With Palatal Involvement. Cureus. 2024;16(3):e57004.

15. Bracamonte JD, Underhill M, Schweda D. Oral Congenital Melanocytic Nevi: A Rare Finding. Consultant. 2023;63(2):e9.

16. Takeda Y. Congenital nevocellular nevus of the oral mucosa. Annals of dentistry. 1988;47(2):40-2.

17. Meng T, Zhang HL, Long X, Wang XJ. Functional and aesthetic reconstruction of a large upper lip defect using combined three local flaps: A case report. Medicine (Baltimore). 2018;97(12):e0191.
